# Use of Cotton Textiles Coated by Ir(III) Tetrazole Complexes within Ceramic Silica Nanophases for Photo-Induced Self-Marker and Antibacterial Application

**DOI:** 10.3390/nano10061020

**Published:** 2020-05-27

**Authors:** Ilaria Zanoni, Magda Blosi, Valentina Fiorini, Matteo Crosera, Simona Ortelli, Stefano Stagni, Alessandra Stefan, Sotiris Psilodimitrakopoulos, Emmanuel Stratakis, Francesca Larese Filon, Anna Luisa Costa

**Affiliations:** 1 Institute of Science and Technology for Ceramics, CNR-ISTEC-National Research Council of Italy, Via Granarolo 64, I-48018 Faenza, RA, Italy; ilaria.zanoni@istec.cnr.it (I.Z.); simona.ortelli@istec.cnr.it (S.O.); anna.costa@istec.cnr.it (A.L.C.); 2 Clinical Unit of Occupational Medicine, Department of Medical and Surgical Sciences, University of Trieste, Via della Pietà 2/2, 34129 Trieste, Italy; larese@units.it; 3 Department of Industrial Chemistry “Toso Montanari”, University of Bologna, Viale Risorgimento 4, I-40136 Bologna, Italy; valentina.fiorini5@unibo.it (V.F.); stefano.stagni@unibo.it (S.S.); 4 Department of Chemical and Pharmaceutical Sciences, University of Trieste, via L. Giorgieri 1, 34127 Trieste, Italy; mcrosera@units.it; 5 Department of Pharmacy and Biotechnology, University of Bologna, Viale Risorgimento 4, I-40136 Bologna, Italy; alessandra.stefan@unibo.it; 6 Consorzio Sistemi a Grande Interfase (CSGI), University of Florence, Via della Lastruccia 3, 50019 Sesto Fiorentino (FI), Italy; 7 Institute of Electronic Structure and Laser, Foundation for Research and Technology-Hellas, N. Plastira 100, 70013 Heraklion, Crete, Greece; sopsilo@iesl.forth.gr (S.P.); stratak@iesl.forth.gr (E.S.); 8 Department of Materials Science and Technology, University of Crete, 71003 Heraklion, Crete, Greece

**Keywords:** cotton textiles, iridium (III) luminescent complexes, silica host matrix, antibacterial properties, singlet oxygen

## Abstract

This study was aimed at the production and characterization of coated cotton textiles with luminescent ceramic nanophases doped with cationic Ir(III) tetrazole complexes. We confirmed that SiO_2_ nanoparticles (NPs) do not affect the phosphorescent properties of the complexes that maintain their emission (610 and 490 nm). For the first time we transferred the luminescence feature from nanosol to textile surface, highlighting the advantages of using nanosilica as an encapsulating and stabilizing matrix. The optimized Ir@SiO_2_ suspensions were homogenously applied onto the cotton surface by dip-pad-dry-cure technique, as proved by the 2p-fluorescence microscope analysis. Once we verified the self-marker properties of the Ir(III) complex, we observed an excellent washing fastness of the coating with a very limited release. SiO_2_ in the washing water was quantified at maximum around 1.5 wt% and Ir below the inductively coupled plasma optical emission spectrometry (ICP-OES) detection limit of 1 ppm. A Franz cell test was used to evaluate any possible ex-vivo uptake of Ir@SiO_2_ nanoparticles across human skin tissues, showing that epidermis and dermis stop over 99% of Ir, implying a reduced impact on human health. The light-induced antimicrobial potential of the Ir@SiO_2_ were assessed toward both Gram(−) and Gram(+) bacteria. The results encouraged further developments of such functional textiles coated by self-markers and antibacterial active nanophases.

## 1. Introduction

The increasing demand for innovative, functional, and smart textiles is one of the major challenges that fabric and clothing industries are requested to face nowadays. In particular, the end user market requires accessible products associated with advanced features such as photocatalytic performance and self-cleaning properties [1,2,3], antimicrobial activity [4,5], thermo and photo-chromism [6], and fire retardancy [7,8,9]. One novel approach pursued to realize this kind of innovative materials involves substrate nano-functionalization with nanoparticles (NPs capable of imparting specific functionalities once anchored to the surface of fabrics). At the same time, the need to easily detect the presence of NPs and monitoring their persistence and distribution is a major concern in product manufacture, not only during the working process but also in its intended use [10]. Therefore, the decoration of NPs with appropriate markers or dyes that can be easily detected by means of fast and non-invasive techniques, would provide a reliable approach to realize innovative fabrics and to monitor their in-use behavior. For this reason, we recently developed phosphorescent systems based on silica nanoparticles (SiO_2_ NPs) doped with luminescent dyes represented by cationic Iridium(III) tetrazole complexes [11,12,13,14]. Silica is well known for its chemical, optical, and thermal inertia, with a proven compatibility with light emission of transition metals-based complexes [15,16]; in addition, it is often marketed in the form of stable colloidal suspensions at an affordable cost [17]. Therefore, silica was selected as the ideal carrier matrix, likely capable of leaving the functional properties of the active luminescent phase unaltered. At the same time, upon their anchoring on silica matrix, the Ir(III) complexes were endowed with enhanced chemical, thermal, and optical stability, leading to composites characterized by a reduced loading of the expensive luminescent markers. Cyclometalated Ir(III) complexes are widely known for their intense and color tunable luminescent output, which is found to span the entire spectrum of visible light as a consequence of slight modifications of their chemical structure. These peculiar photoluminescent performances have prompted the use of Ir(III) cyclometalates as emitters in lighting devices, and as suitable luminescent bioprobes [12,18,19,20]. In addition, compared to Ru(II) and many other classes of metal-based derivatives, the antibacterial activity of Ir(III) complexes have been less explored. For this reason, promising antibacterial activities of a set of cationic cyclometalated complexes towards Gram(+) bacteria (e.g., *Staphylococcus aureus* and *Deinococcus radiodurans*) [13,21,22,23] was recently demonstrated, opening further chances of applications for this class of luminescent compounds. 

However, to finalize their application as textile coatings, the possible toxicity of Ir(III) complexes for human skin (the final biological target) must be evaluated. Iridium (III) complexes can have non-negligible cellular toxicity and are water insoluble, resulting in poor biocompatibility and limited biomedical application [24,25]. However, Ir(III) complexes can be used as an anticancer drug [26]. Recently, Zhang et al. (2019) suggested the use of red-emitting Ir (III) complex displaying aggregation-induced emission for photodynamic therapy [27]. Ir(III) complexes can be considered as ideal photosensitizers, thus opening the path to interesting applications in cancer therapy, even though their mechanism of action is not fully understood [28,29,30].

We have here identified two cationic Ir(III)-tetrazole complexes, [F_2_IrPTZ-Me]^+^ and [IrQTZ-Me]^+^, characterized by different luminescent properties and we investigated their self-marking properties for silica nanoparticles (Ir@SiO_2_ NPs) [14], relating their optical properties with antibacterial effects once embedded inside the matrix used as a cotton coating medium. The results reported herein highlight switching on of their antimicrobial activity toward both Gram(+) (*Deinococcus radiodurans*) and Gram(−) (*Escherichia coli*), upon embedding in silica matrix (Ir@SiO_2_ systems) and on cotton textiles. After discovering the advantages of nanosilica as encapsulating matrix for Ir complexes [14], here for the first time we moved forward, advancing the applicability of Ir complexes in the form of coatings. To this end, a step by step material design allowed us to transfer the luminescence feature from nanosol to textile surface in the form of coating. Silica nanoparticles were considered as carriers to improve the dispersion of Ir(III) complexes in suspension, otherwise unfeasible and then facilitating their further application as textile coating. 2p fluorescence microscopy and spectrofluorimetry confirmed the coating homogeneity on the cotton surface, while the low release detected after washing fastness test pointed out its optimal attachment. The potential toxicity of Ir(III) markers in terms of possible penetration of Ir@SiO_2_ NPs through human skin was investigated using a Franz cell test, as a consequence risk assessment and management were evaluated to avoid any health damage to workers and users exposed to coated textiles.

## 2. Materials and Methods

### 2.1. General Considerations

All the reagents and solvents were purchased by Merck (Kenilworth, NJ, USA), Alfa Aesar (Kandel, Germany), Strem Chemicals (Bischheim, France) and applied without any additional modification. Silica nanosuspension (Silica LUDOX^®^ HS-40) was purchased by Grace Davison (Columbia, MD, USA) and treated by means of an exchanging resin (Dowex 50x8 protons, Merck, Mesh 20-50, Kenilworth, NJ, USA). Ir(III) complexes were purified by column chromatography, using Al_2_O_3_ as the stationary phase. Electrospray ionization (ESI)-mass spectra were acquired using a Waters ZQ-4000 instrument (Waters, Milford, MA, USA) and nuclear magnetic resonance spectra (^1^H and ^13^C) were acquired using a Varian Mercury Plus 400 (Varian, Palo Alto, CA, USA). As textile substrate we used a 260 g/m^2^, 98% cotton fabric and 2% EA elastomer containing about 85% polyurethane.

### 2.2. General Procedure for the Preparation of the Cationic Ir(III) Complexes

**Scheme 1:**
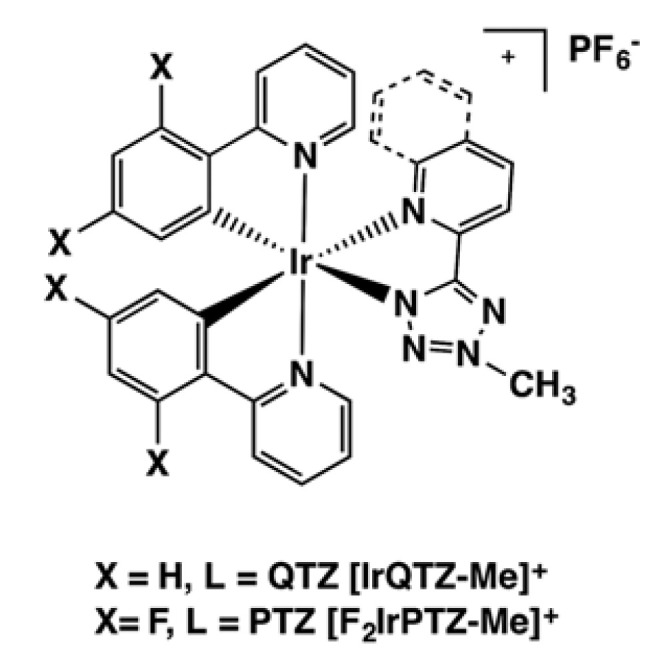
Molecular structure of cationic Ir(III) tetrazole complexes.

We prepared the cationic Ir(III)-complexes following the procedures described in our previous work [13]. The molecular structures are reported in Scheme 1.

### 2.3. General Procedure for the Colloidal Physical Encapsulation

The Ir(III)-tetrazole based nanosols were encapsulated following a procedure that was optimized in our previously reported work [14]. 

### 2.4. General Procedure for the Dip-Pad-Dry-Cure Process for Fabrics

Fabric samples, size 7 × 7 cm^2^, were washed in an ultrasonic bath for 30 min (15 min with soap and water, and 10 min with water). The fabric coupons were immersed into the two different Ir@SiO_2_ suspensions (3 wt%) and soaked for 5 min, then they were squeezed by means of a two-rollers laboratory padder, dried in oven at 100 °C, and finally heat treated for 10 min at 130 °C. Three impregnation steps were performed to reach the desired dry add-on value (AO%) and the fabric coupons were heat treated at 100 °C for 5 min after each deposition step. The AO% was described as increase of the sample weight, due to the coating, and was calculated as previously reported by Ortelli et al. (2015) [1].

### 2.5. Colloidal Characterization

Colloidal characterization was reported and performed in a previous work [14]. Dynamic light scattering and electrophoretic light scattering analysis on the nano-dispersions were performed using a Zetasizer Nano instrument ZSP, ZEN5600, (Malvern Instruments, Malvern, UK). The evolution of the Zeta potential along the pH range for the Ir@SiO_2_ systems was followed by means of acid and basic titrations using a Zetasizer Nano instrument coupled with an automatic titrating system. 

### 2.6. Morphological Characterization of Fabric Samples

The morphological characterization of pristine and Ir@SiO_2_-coated cotton fabrics was carried out by electron microscopy measurements using a field emission scanning electron microscope, SEM-FEG (FEI Magellan 400L SEM High Resolution SEM, Hillsboro, OR, USA). The uncoated and coated cotton fabric coupons (about 3 mm × 3 mm) were anchored to a carbon conductive sticky tape and gold sputtered or silver pasted. 

### 2.7. Photophysical Characterization

Absorption spectra were measured at room temperature (RT) by Perkin Elmer Lambda 35 UV/vis spectrometer (PerkinElmer, Waltham, MA, USA). Emission of uncorrected steady-state and excitation spectra were measured by Edinburgh FLSP920 spectrometer. Both emission and excitation spectra were cut-off using a filter (395 nm) and automatically adjusted with an internal calibration curve by the instrument. Experimental deviations are evaluated to be ±8% for lifetime determinations, ±20% for quantum yields, ±2 nm for absorption peaks, and ±5 nm for emission peaks. More details on the photophysical investigations and characterization were reported in the Supplementary Files document [31,32]. The dispersion homogeneity of the complexes within the silica matrix, once deposited as coating, and textiles tomography were acquired by a 2p-fluorescence microscope as described in detail by Psilodimitrakopoulos et al. (2018) [33]. Briefly, the setup was based on a *fs* excitation laser source, centered at 1030 nm, with ~1 W output power and 76 MHz repetition rate (Pharos-SP, Light Conversion, Vilnius, Lithuania), and a 20x/0.8NA objective (Carl Zeiss, Jena, Germany) and emission sample was acquired with a bandpass filter (620 ± 52 nm, Semrock, Rochester, NY, USA) for Ir@SiO_2_ systems. Qualitative analysis on images acquired by 2p-fluorescence microscope was done using ImageJ 1.52p software (National Institute of Health, Bethesda, MD, USA).

### 2.8. Washing Fastness Test

The Ir(III) complexes and silica NPs’ release from Ir@SiO_2_-coated cotton samples was performed under static and dynamic conditions to simulate washing cycles of the fabric while in use and to study adhesion to the substrate and quantify the weight loss. The dynamic test was performed by dipping the coated cotton sample (size 5 × 5 cm^2^) in 25 mL MilliQ water bath and ultrasonicating it for three cycles, 10 min each, at RT. After every cycle, the washing water was stored and analyzed at inductively coupled plasma optical emission spectrometry (ICP-OES). At the end of each cycle, the sample was dried in the oven at 80 °C for 10 min and washed again. The static test was performed by immersing the samples (size 7 × 7 cm^2^) in a MilliQ water bath (50 mL) at RT for to ten days and sampling washing water after one, four hours and one, three, four, seven, and ten days. For the dynamic test we collected data from coated textiles, from silica-coated textiles, and from samples coated with the two Ir@SiO_2_ complexes. Release under static conditions involved only the blue Ir@SiO_2_-coated substrate as reference. Measurements of the collected washing waters were performed by inductively coupled plasma optical emission spectrometry using an ICP-OES 5100—vertical dual view apparatus (Agilent Technologies, Santa Clara, CA, USA) to determine SiO_2_ and Ir@SiO_2_ complex content. The analysis was performed using radial viewing mode, at a viewing height of 8 mm, and calibration curves were carried out with 0.1, 0.5, 5.0, 10.0, and 100.0 mg·L^−1^ standards for all analyzed elements. In order to digest any released particles nitric acid (65%-Titolchimica) was added to both standards and samples in 1:10 in volume ratio. Finally, all calibration curves were assessed, finding an *R*^2^ correlation coefficient >0.99. The concentration of SiO_2_ in washing water was evaluated by ICP-OES Si determination, while the release of Ir(III) complex was analyzed taking the Ir amount as reference.

### 2.9. Bacterial Strains and Media

*Escherichia coli* (*E. coli*), Gram-negative bacterium (TOP10 strain-Invitrogen), was grown in Luria–Bertani (LB) medium (0.5% yeast extract, 1% NaCl, 1% tryptone). To obtain the solid plates, 1.5% of agar was added to the culture medium. *Deinococcus radiodurans* (*D. radiodurans*), a red-pigmented Gram-positive bacterium, was cultivated in TGY medium (0.1% glucose, 0.3% yeast extract, 0.5% trypton), if necessary, we added 1.5% of agar.

### 2.10. Antibacterial Evaluation

Antibacterial performances of Ir@SiO_2_-coated textiles against *E. coli* and *D. radiodurans* were evaluated using the antibacterial activity assessment for textile materials, the Parallel Steak Method (AATCC 147 modified) procedure [34]. A dilution of a liquid overnight culture (0.1 mL, for a concentration corresponding to approximately 1 × 108 CFU·mL^−1^) was streaked on LB and TGY agar plates, producing bacterial lawns in parallel streaks. Non-sterile samples of blue and red Ir@SiO_2_-coated fabric are cut into 25 × 50 mm^2^ pieces. These fabric samples are located on the top of the inoculate agar plates and incubated at 30 ± 2 °C for 24 h. We compared three different lighting scenarios: darkness, exposed to lab light, and exposed for 45 s to UV light (*λ*_max_ = 365 nm) and then to lab light for the 24 h of exposure. After the incubation step, the agar plates were examined to check the possible interruption of bacterial growth under the textile sample and the presence of inhibition zone beyond the sample edge. If no bacterial colonies are observed under the textile sample with the presence of an inhibition zone, we considered the antibacterial activity as acceptable. AATCC 147 modified method cannot be taken into consideration as a quantitative test, but it can be used to identify the presence of an antibacterial activity among different samples. We have used resistant *E. coli* and *D. radiodurans*, as our standard test cultures.

### 2.11. Preparation of Skin Membranes

The skin membrane preparation follows the procedure reported in our previous studies (Crosera et al. 2018 and Zanoni et al. 2019) [35,36]. Samples were collected from skin waste of human abdominal surgery, as approved by the Trieste Hospital Ethical Committee n° 236/2007. Subcutaneous fat in full thickness human skin was removed and hair shaved, then all samples were frozen and stored at −25 °C for a period of a maximum of 4 months [37]. The integrity of skin samples was evaluated using the trans epidermal water loss (TEWL) method, as described by Guth et al. (2015) [38]. Samples, that presented a TEWL value >10 g·m^−2^·h^−1^, were considered damaged and unsuitable for the test. 

### 2.12. In Vitro Diffusion System

Static diffusion cells were used to study the transdermal passage of Ir@SiO_2_ systems through human skin following the Franz method (1975) [37]. The Franz cell was structured in three compartments that mimic the dermal exposure: the donor (where exposure material is located), the skin membrane, and the receptor one. The receptor compartment presents a chamber (mean volume of 14.0 mL) that is continuously magnetically stirred and thermostated at 32 °C by the jacket surrounding the cell, in order to mimic the usual hand’s physiological temperature value. The salt concentration in the receptor fluid reproduces roughly the blood condition. Each skin sample was clipped between the donor and the receptor compartment; for each skin sample in the cell the mean exposed area was 3.3 cm^2^ and the average thickness was 1 mm. 

The experiments were performed as reported hereinafter. At time 0, the donor chambers of four Franz cells were loaded with 18 mg·cm^−2^ of blue or red Ir@SiO_2_ NPs solution dispersed in water (conc. 3% w/w) and diluted 1:2 with synthetic sweat (4 mL solution in donor compartment), reproducing the in vivo conditions typically at pH 4.5. Two blank cells were obtained without introducing the Ir@SiO_2_ dispersion and filling the exposure chamber with synthetic sweat. After 24 h, the receiving phases were collected to perform the concentration analysis and the skin samples removed, divided into epidermis and dermis by dipping them in hot water (60 °C) for one minute (heat shock), and acid digested by microwave (MultiwavePRO, Anton Paar, Graz, Austria). 

Ir (mass selected: 193 a.m.u.) concentration, in the receptor fluid and in the solutions derived from the digestion of the skin membranes, was measured by an inductively coupled plasma mass spectrometry (ICP-MS). The instrument used for the analysis was a NexION 350 X (PerkinElmer, Waltham, MA, USA). The samples’ concentration was obtained by means of standard calibration curves (0.01, 0.1, 1, and 10 µg·L^−1^). The variation coefficient for the Ir detection was always below 5%. 

## 3. Results and Discussion

### 3.1. Synthesis of Luminescent Ir@SiO_2_ Nanosols Systems

In order to accomplish the functionalization of negatively-charged SiO_2_ NPs, two cationic cyclometalated Ir(III) tetrazole complexes were selected as phosphorescent emitters. The choice of considering these specific compounds, namely [F_2_IrPTZ-Me]^+^ and [IrQTZ-Me]^+^, is motivated by them displaying markedly different emission colors. Indeed, the [F_2_IrPTZ-Me]^+^ presents a luminescent output centered in the blue region (*λ*_max_ = 490 nm), while the complex [IrQTZ-Me]^+^ emits at *λ*_max_ = 630 nm in the red region of the visible spectrum [13]. The complexes were prepared by means of a procedure developed by our recent work [13]. The main results coming from their characterization are reported in Supplementary Information (Figures S1–S6). The iridium (III) complexes were physically adsorbed on colloidal SiO_2_ NPs, which act as encapsulating matrix. Silica interaction does not affect the phosphorescence of the complex (Table S1 and Figure S7). As reported in our previous work [14], Ir@SiO_2_ were performed and optimized with the aim of obtaining self-marker products that emit in the blue and red regions of visible light (blue- and red-Ir@SiO_2_, due to the presence of [F_2_IrPTZ-Me]^+^ and [IrQTZ-Me]^+^, respectively).

### 3.2. Ir@SiO_2_ Nano-Coated Textiles

**Figure 1:**
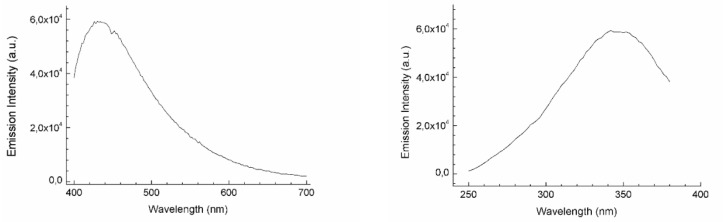
(**left**) Emission and (**right**) excitation profiles of untreated cotton textile.

**Figure 2:**
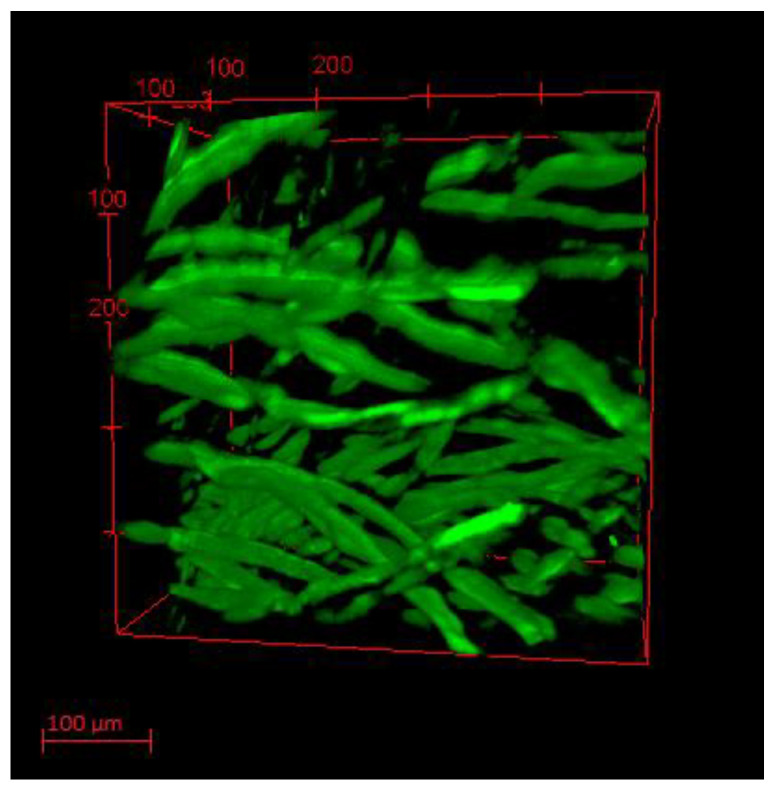
Tomography image of cotton textile (350 µm depth) made by 2p-fluorescence microscope.

After optimization of dispersed Ir@SiO_2_ sols [14], we investigated their application in the field of markable nano-functionalized textiles. To this end, the luminescent Ir@SiO_2_ sols were anchored onto cotton textiles using the dip-pad-dry-cure technique, with thermal treatment in an oven used to strengthen the coating bond to the substrate. Since the presence of terminal hydroxides on SiO_2_ NPs allows interaction with a large number of –OH groups on the surface of each fiber, the cellulose chain of cotton fibers is particularly well suited for this work’s purposes [1]. As with most commercial cotton fibers [39], the commercial textiles samples used in this work contain an optical brightener, the presence of which is responsible for the emission centered at ca. 430 nm (Figure 1). Exploiting two-photon (2p) excited fluorescent emission of such dyed cotton, we are able to reproduce the textile 3D structure, using a 2p-fluorescence microscope and analyzing the first 350 µm of the surface, as shown in Figure 2. 

Moreover, the emission from the optical brightener partially overlaps with the emission of Ir@SiO_2_ systems in the blue region of visible light. In order to ensure the optimal adhesion on cotton together with a noticeable emission typical of the Ir@SiO_2_ systems, we performed three impregnation steps on the treated textiles. The percent increment values, hereinafter referred as the % add-on, showed a linear correlation between number of layers, nanosol concentration, and solid content weight of the textiles (Table 1). In fact, the obtained add-on values after three impregnation layers are about threefold the nanosol concentration values (3 wt%) without any effect induced by the iridium complex on the adhesion (Table 1), thus confirming a strong interaction between the nanostructured coating and the substrate [1]. 

**Figure 3:**
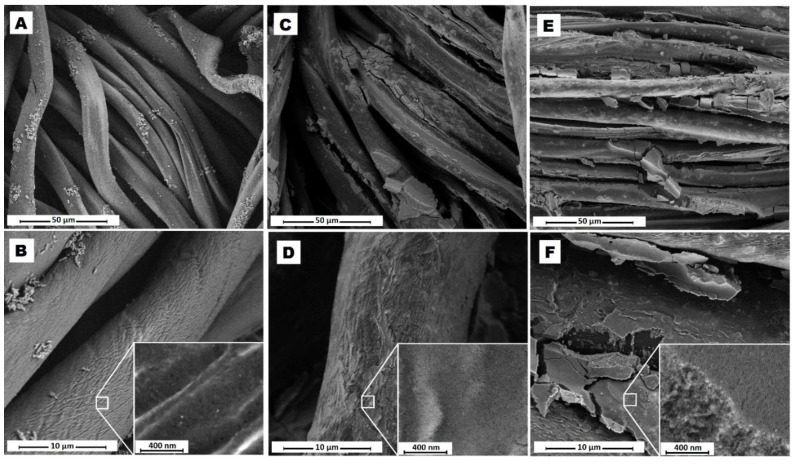
SEM-FEG images of untreated cotton textile (**A**,**B**), blue Ir@SiO_2_-coated textiles (**C**,**D**), red Ir@SiO_2_-coated textiles (**E**,**F**) at different magnification.

To investigate the coating distribution on the substrate, SEM-FEG surface images were acquired both from untreated and Ir@SiO_2_-coated samples. After two washing cycles with MilliQ water, the cotton fibers of the untreated sample (see Figure 3A,B) still appear well organized and clean, except for small amounts of calcium carbonate CaCO_3_, whose detection, is consistent with a residual MilliQ water contaminant. At higher magnification it is possible to distinguish a characteristic superficial micro porosity of the cotton fiber, useful for the successive impregnation step (Figure 3B). On the other hand, as shown in Figure 3C–F, the two Ir@SiO_2_ nanosol systems applied on cotton textiles cover all the fabric area, filling the inter-fiber space and promoting a so-called *ceramized coating*. The primary nanosized spherical particles, constituting the coating, can be observed at higher magnification (Figure 3D–F). EDX spectrum confirmed the presence of Si on the whole cotton area, on the other hand Ir signal was not well resolved because the Ir amount is too low to be detected and in addition its peak overlaps with the Si-K signal (Figure S8). 

**Figure 4:**
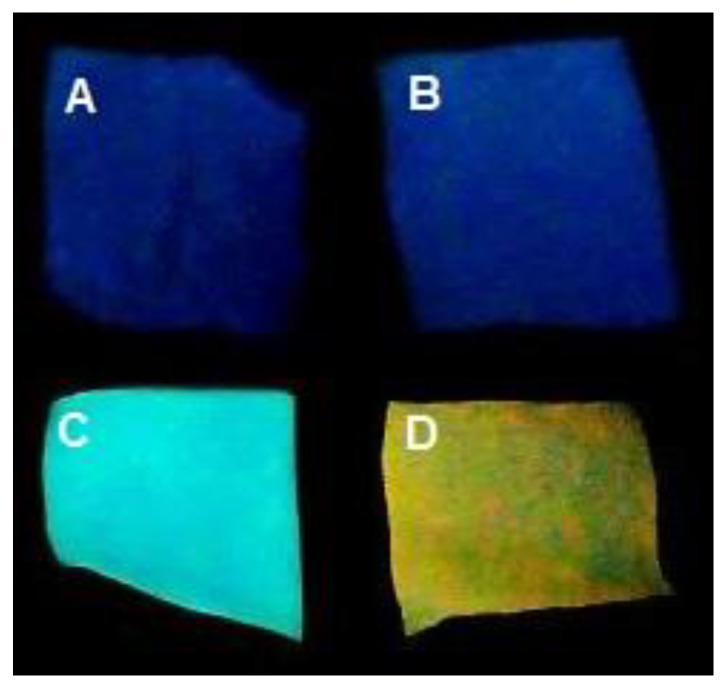
Real image of fabric samples under UV lamp (*λ*_em_ = 365 nm): uncoated (**A**), silica coated (**B**), blue Ir@SiO_2**-**_coated (**C**) and red Ir@SiO_2**-**_coated (**D**) cotton textiles.

**Figure 5:**
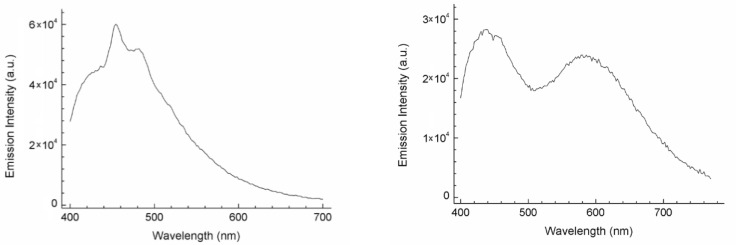
Emission profile of cotton textile coated by blue Ir@SiO_2_ system (**left**) and by red Ir@SiO_2_ system (**right**).

Concerning the luminescent properties of the substrates after three treatment cycles, the data point out that all the prepared samples were qualitatively traceable under UV lamp (*λ*_em_ = 365 nm), showing an unchanged emission of Ir(III) complexes, with good dispersion of the complex into the silica matrix (Figure 4). Besides luminescence Ir@SiO_2_ coating imparts new functional properties to the treated textiles, such as hydrophilicity and abrasion resistance provided by the silica matrix. Luminescent emission from this kind of substrate represents an especially difficult goal to achieve, due to a liquid-to-solid state transfer of the chromophore molecules and to the presence of optical brightener, whose optical emission compete with and partly overlap the complexes’ emission. However, by means of three-layer impregnation we were able to clearly distinguish the emission contribution of the complexes from that of the optical brightener. In this way, samples can be observed directly under the UV lamp, whereupon untreated and treated samples appear clearly different (Figure 4), maintaining the expected luminescent output. In particular, the corresponding emission spectra of the treated samples result in broad spectral curves from the overlapping of Ir complex and brighter dye spectral profiles (Figure 5). Since the blue Ir@SiO_2_ system emits in the same visible range of the optical brightener, the resulting spectrum can roughly be considered as the sum of the two contributions without shifts in their emission maxima. In contrast, this drawback is not observed for the system based on [IrQTZ-Me]^+^, i.e., red Ir@SiO_2_. Indeed, the emission profile of red Ir@SiO_2_ was shifted from 610 nm for the nanosol state to 590 nm as a coating, due to the typical ^3^MLCT-based emission evidenced also by the noticeable rigidochromic blue shift encountered at 77K (Figure S9). As well, in passing from a nanosol dispersed system to a nanosol coating, the complex structure increases its stability due to silica embedding and solid state transfer, causing rigidochromic blue shift [18,20,40,41,42].

**Figure 6:**
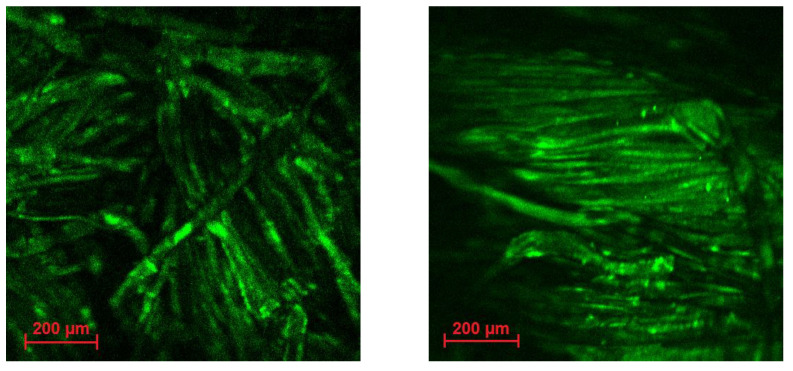
2p-fluorescen image of textiles coated by blue Ir@SiO_2_ system (**left**) and red Ir@SiO_2_ system (**right**).

Taking advantage of the emission resulting from Ir(III) complexes, 2p-fluorescence microscopy was used to detect both distribution and homogeneity of Ir@SiO_2_ systems on textiles. As shown in Figure 6, it is possible to reconstruct each single fiber of the fabric—detecting only the complex emission—by cutting off the optical brightener contribution with a 620 ± 52 nm bandpass filter. The results evidenced that the complexes coupled and vehiculated by the silica matrix covered all the fibers in a homogeneous way by means of H-bonds established between SiO_2_ and the cellulosic hydroxyl groups. Using qualitative analysis by ImageJ software, we compared samples with and without the luminescent complexes. As expected, Ir@SiO_2_ displayed much brighter emissions than both plain cotton and silica-coated cotton (Figure S10). In fact, by evaluating the color intensity of the images collected by fluorescence microscopy, in the presence of the Ir@SiO_2_ complexes, we detected an increase of intensity by more than 1 × 10^6^ counts. 

### 3.3. Washing Fastness

**Figure 7:**
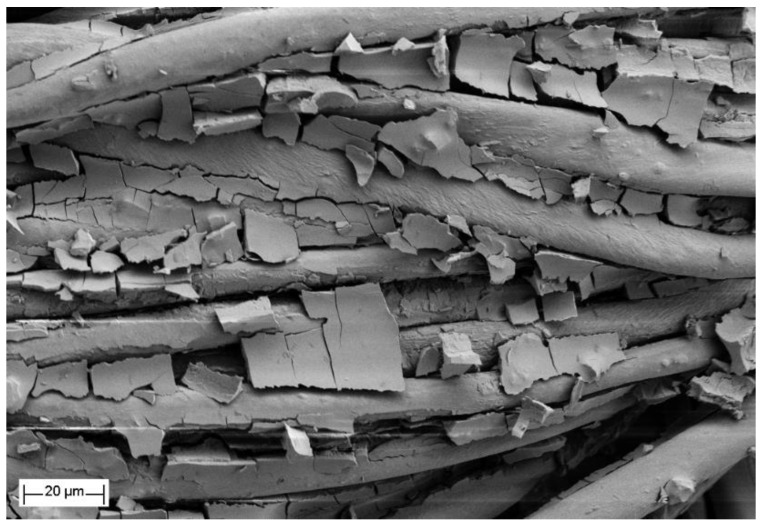
SEM-FEG images of textiles coated by three-layer of blue Ir@SiO_2_ system after 10 days of washing test in static condition.

Coating adhesion and washing fastness were evaluated by exposing the textiles to washing tests in dynamic and static conditions [4], with the intent to reproduce the fabrics behavior during washing and in use stages. The washing fastness tests followed a constant methodology for ICP-OES analysis. The obtained values are negligible both in static and dynamic condition, reaching at most only some units percent for all the systems, without any detectable release of Ir(III) complexes (Figure S11 and in Tables S2 and S3) [43]. In particular, dynamic tests results showed no significant variation in the behavior of silica or Ir@SiO_2_ coatings as to washing fastness, giving similar values of total silica weight loss (ca. 2%). After 4 days in static conditions, silica release flattened out, stabilizing at ca. 1.5% weight loss. These results indicate strong adhesion of the coating and good embedding of the Ir(III) complexes into the SiO_2_ matrix, which also promotes a strong interaction with the fibers. SEM-FEG image (Figure 7 and Figure S12) collected on the blue Ir@SiO_2_-coated textile sample after washing confirmed the persistence of the coating homogenously distributed on the cotton fibers, further proved by the EDX Si signal detection on the cotton area and finally consistent with the excellent washing fastness behavior. In addition, the luminescence durability of the coating was checked, proving to be long lasting. In fact, the same luminescent grade was kept even after 2 years from preparation (Figure S13C,D) and after the washing tests (Figure S13B).

### 3.4. Antibacterial Properties

**Figure 8:**
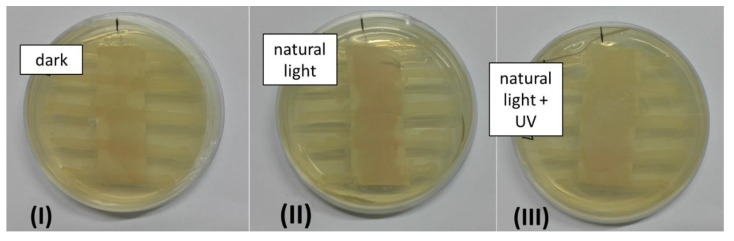
Image of AATCC 147 tests on blue Ir@SiO_2_-coated textiles in the dark (**I**) and under natural light (**II**) and natural light + UV (**III**) conditions.

The antibacterial properties of Ir(III)-based luminescent textiles was compared to those displayed by [F_2_IrPTZ-Me]^+^ and [IrQTZ-Me]^+^ alone against Gram(−) *E. coli* and Gram(+) *D. radiodurans*. As previously reported, both of the cationic Ir(III) tetrazole derivatives promoted excellent antibacterial activity toward Gram(+) *D. radiodurans* (MIC = 1 μg·mL^−1^ for [F_2_IrPTZ-Me]^+^, 4 μg·mL^−1^ for [IrQTZ-Me]^+^), while no antimicrobial activity was observed for Gram (−) *E. coli* cultures [13]. According to the procedure reported in the antibacterial activity assessment of textile materials—parallel steak method (AATCC 147, modified)—the antibacterial activity of coated textiles in direct contact with agar cultures was tested [34]. This test was chosen as a qualitative method to compare any differences in their antibacterial strength between Ir(III) complexes alone and Ir(III) complexes embedded inside the silica coatings. After an incubation phase of 24 h at 30 °C, three different exposure conditions were analyzed: dark, natural light, and UV + natural light. Bacterial inhibition is usually associated with an alteration of the parallel streaks marked out on the agar plates corresponding to bacterial colonies. Unlike results reported by Fiorini et al. (2017) [13], we observed for both [F_2_IrPTZ-Me]^+^ and [IrQTZ-Me]^+^ complexes encapsulated within silica as coating and exposed to visible light an antimicrobial activity against *E. coli* cultures. Indeed, *E. coli* growth was inhibited by both blue and red Ir@SiO_2_ to a comparable extent (blue Ir@SiO_2_ shown in Figure 8, while for red Ir@SiO_2_ agar plates are shown in Figure S14). *E. coli* growth inhibition was observed on the coated textiles once exposed to natural light and UV + natural light, while no alteration of the streaks is observed for samples left in darkness. These results might suggest a photo-induced antibacterial activity of the Ir(III) triplet emitters (^3^T) once stabilized by silica, an effect that can be correlated with the production of singlet oxygen (^1^O_2_) that—together with other ROS (reactive oxygen species)—might be potentially toxic for bacteria. In fact, singlet oxygen can easily oxidize organic matter, breaking the bacterial membrane with a consequent antimicrobial effect [44,45]. In addition, the activity against Gram(−) highlights that Ir complexes enhance their antibacterial power once stabilized by silica matrix. We hypothesize that the inorganic matrix, enabling a better stabilization of the complex structure, could maximize the efficiency of singlet oxygen production.

These qualitative results pave the way to further hypothesis about the driving mechanism causing the antibacterial activity displayed by Ir@SiO_2_ systems, in which the strong interaction between silica matrix and complex probably plays a key role.

### 3.5. Wearable-Release

With the aim of investigating the coated textiles’ behavior during their usage, we evaluated Ir@SiO_2_ NPs release in static condition under exposure to synthetic sweat [46], mimicking the conditions of sweaty fabric in contact with the human skin, and evaluating any possible release of the final material when wearied. We performed 24-h release tests in static condition at 32 °C, the typical external temperature of human skin [47]. After ICP-OES evaluation, we observed a very low release of SiO_2_ and an Ir signal either below or close to the instrumentation limit of detection (LOD) (Table 2), a negligible value compared with the total amount of coating.

**Figure 9:**
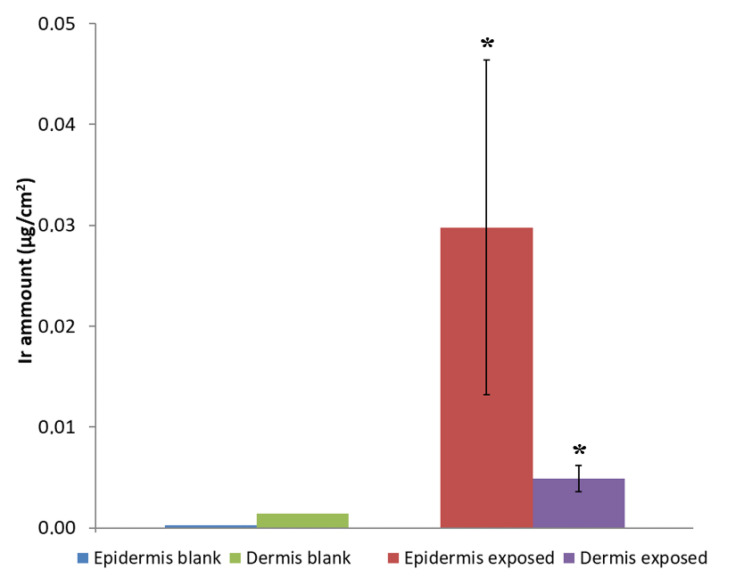
Ir distribution within human skin in blank and treated samples as determined by ICP-MS. (* values are significant different from the blank with *p* < 0.05).

Taking advantage of the Franz cell ex-vivo model, trans-dermal diffusion of Ir@SiO_2_ NPs was evaluated. This choice was made in order to enhance skin exposure by putting it in direct contact with the Ir(III) nanosols instead of sweaty fabric, which showed such a low release of NPs. After 24 h of exposure to Ir@SiO_2_ NPs, no trans-dermal diffusion was observed to occur as confirmed by ICP-MS analysis, which showed no detectable amounts of Ir inside the receiving phases (LOD for Ir^193^ = 0.010 µg·L^−1^). By means of separation of the skin into its epidermic and dermal components, epidermal ad dermal NPs penetration could be evaluated. Analyses detected little penetration of Ir into the dermis, while the amount detected within the epidermis was found to be higher because epidermis acts as the main barrier [48,49]. Typically, the amount of toxic agents detected in the epidermis displays a high variance due to specific donor skin characteristics, such as number of hair bulbs and thickness (Figure 9).

**Figure 10:**
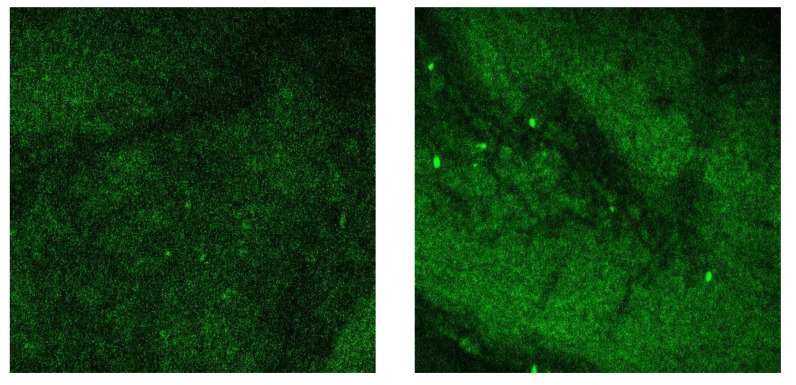
Superficial images made using a 2p-fluorescence microscope of skin sample (**left**) before Ir@SiO_2_ NPs exposure and (**right**) after 24 h of exposure. Image size is 500 µm × 500 µm.

**Figure 11:**
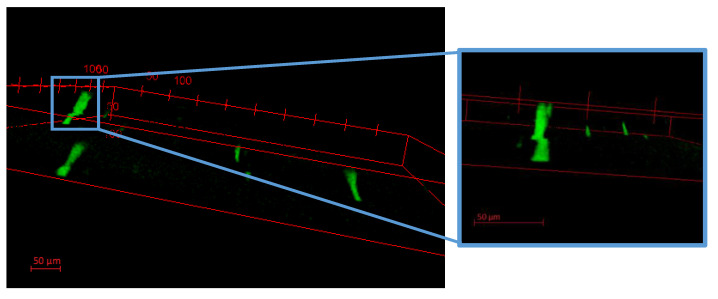
Tomography images made using a 2p-fluorescence microscope of skin sample (1 mm × 1 mm × 50 µm) after 24 h of exposure to Ir@SiO_2_ NPs.

The Ir@SiO_2_ NPs spreading on the epidermal surface was then mapped with 2p-fluorescence microscopy. Moreover, with the use of a (620 ± 52 nm) bandpass filter, this technique enhances Ir(III)-based emission by cutting the emission from the first skin layer, thus collecting only the signal due to NPs. Observing skin samples before and after NPs exposure (Figure 10), we noticed the presence of light dots in the treated sample. A closer z-stack image analyses obtained by acquiring the first 50-µm layers in thickness (Figure 11), revealed luminescent dots that can be associated to the external part of hair channels, having dimensions about 20 µm in diameter. Images seem to confirm the behavior reported in literature for silica NPs with diameter lower than 45 nm [48]. Particularly, Ir@SiO_2_ NPs hypothetically released by the coated textile are stopped in the first skin layers (mainly in epidermis), reaching the hair bulbs and being stuck in them, without any strong adverse effect of the inner skin layers and tissues underneath.

However, in order to reach an optimal risk assessment and management, it’s necessary to identify the real exposure conditions of the fabrics coated with Ir@SiO_2_ systems. 

For both [F_2_IrPTZ-Me]^+^ and [IrQTZ-Me]^+^ cationic complexes we consider 21 µM (15 µg·mL^−1^) and 23 µM (15 µg·mL^−1^), respectively, to be the maximum possible exposure. This estimate is based on the consideration that in the case of direct contact with Ir complexes aided by skin/fabric friction in the presence of sweat, the entire amount of complex embedded in the 3 wt% Ir@SiO_2_ systems (3 g·L^−1^ of Ir@SiO_2_) can be freed and made available for cell uptake. The w/w ratio between Ir(III) complex and SiO_2_ nanosol being 1:1000, dispersion in synthetic sweat (dilution of 1:2) gives a 15 µg·mL^−1^ maximum concentration of complex.

Although IC_50_ values are not available for [F_2_IrPTZ-Me]^+^ and [IrQTZ-Me]^+^ for keratocyte cell line, i.e., the cell line used as a model for human skin, we can consider IC_50_ of other Ir(III) complexes belonging to the same organometallic class reported in the literature, having IC_50_ values in the range of 2–10 µM [50,51,52].

As demonstrated above in the washing fastness section, a quite low release of Ir@SiO_2_ from textiles was detected. So, the only risk could be associated to the Ir@SiO_2_ nanosol, and—as proved by the Franz cell test—we did not observe any trans-dermal migration for both Ir@SiO_2_ systems, which stop in the first layers of the human skin. In fact, based on the permeation values assessed by ICP-OES, epidermis and dermis are able to stop over 99% of Ir due to crowding around hair bulbs. Therefore even assuming the maximum possible release under friction-aided conditions, with all of the Ir(III) tetrazolate complexes free and available, the final short-term exposure should be much lower than the average IC_50_ values (2–10 µM) reported in the literature. In conclusion, our final biological target, i.e., the keratocyte cell line, presents a very low exposure risk to Ir(III) tetrazolate complexes, whose concentration in the skin should be negligible. However, more long-term exposure and cytotoxicity tests will be needed for achieving the safe industrial production and use of Ir@SiO_2_-coated textiles.

## 4. Conclusions

We obtained multifunctional luminescent textiles realized by applying for the first time new Ir@SiO_2_ compounds in the form of coating. Treated textiles displayed very promising self-marking properties together with antibacterial activity. The strong interaction between Ir complexes and silica nanoparticles gives these systems the ability to be easily detected under UV light, both when used as coating for textiles and after release in the environment. The homogeneous distribution of such luminescent coating can be usefully exploited as a products’ wear diagnostic tool. In addition, the antibacterial testing shows interesting light-induced antimicrobial properties against both Gram(+) (*Deinococcus radiodurans*) and Gram(−) (*Escherichia coli*), probably due to a reactive oxygen species production. Finally, a preliminary human dermal exposure assessment candidate such Ir@SiO_2_ coating as a safety by design solution able to be easily tracked during its whole life cycle, from nanoparticles production to their release.

## Figures and Tables

**Table 1:** Add-on per cent values evaluated for three layers coated textiles.

Coated Textile	SiO_2_ *	Blue Ir@SiO_2_	Red Ir@SiO_2_
% add-on	7.9 ± 0.5	8.0 ± 0.7	8.9 ± 1.0

* Commercial SiO_2_ destabilized by means of treatment with cationic-exchange resin.

**Table 2:** Release values in synthetic sweat of Ir@SiO_2_-coated fabric after 24 h in static condition at 32 °C.

Release in Synthetic Sweat	Ir (mg·L^−1^)	Si (mg·L^−1^)	Weight Loss of SiO_2_%
Ir@SiO_2_ NPs	0.01 ± 0.01	7.92 ± 0.16	0.95 ± 0.01

## Supplementary Materials

The following are available online at https://www.mdpi.com/2079-4991/10/6/1020/s1, Figure S1: ^1^H NMR of [F_2_IrPTZ-Me]^+^, CD_3_CN, 400 MHz; Figure S2: ^1^H NMR of [IrQTZ-Me]^+^, CD_3_CN, 400 MHz; Figure S3: ^13^C NMR of [F_2_IrPTZ-Me]^+^, CD_3_CN, 400 MHz; Figure S4: ^13^C NMR of [IrQTZ-Me]^+^, CD_3_CN, 400 MHz; Figure S5: ESI-MS spectrum of [IrQTZ-Me]^+^ (positive ions region) [M]^+^ = 712 *m/z*, CH_3_CN; Figure S6: ESI-MS spectrum of [F_2_IrPTZ-Me]^+^ (positive ions region) [M]^+^ = 734 *m/z*, CH_3_CN; Figure S7: Comparison between normalised emission profiles [F_2_IrPTZ-Me]^+^ (blue line) and [IrQTZ-Me]^+^ (red line) complexes and their Ir@SiO_2_ systems (black lines); Figure S8: EDX spectrum of blue Ir@SiO_2_ coated textile; Figure S9: Comparison between normalised emission profile of red Ir@SiO_2_ nanosol system (black) and once used as coating on cotton textile (red); Figure S10: Qualitative evaluation of 2p-fluorescen images made by ImageJ on untreated cotton textiles and samples of cotton textile coated by silica, blue Ir@SiO_2_ system and red Ir@SiO_2_ system; Figure S11: Release tests results collected both in dynamic (left) and static condition (right) for textiles coated by three-layer of blue Ir@SiO_2_ system; Figure S12: EDX spectrum of blue Ir@SiO_2_ coated textile; Figure S13: Real image of fabric samples under UV lamp (λ_em_ = 365 nm): silica coated (A), blue Ir@SiO_2_ coated after 10 days of washing test in static condition (B), blue Ir@SiO_2_ coated after 2 years (C) and red Ir@SiO_2_ coated after 2 years (D) cotton textiles; Figure S14: Image of AATCC 147 tests on red Ir@SiO_2_ coated textiles once exposed in dark (I), natural light (II) and natural light + UV (III) conditions; Table S1: Spectrometric relevant data, expressed as absorption and emission, of cationic Ir(III) complexes and Ir@SiO_2_ nanosols systems; Table S2: SiO_2_ weight loss% data relative to release test in dynamic condition (test for three layers coated textiles); Table S3: Cumulative SiO_2_ weight loss% data relative to release test in static condition for blue Ir@SiO_2_ coating (test for three layers coated textiles).
