# Supplementary material for: Use of Cotton Textiles Coated by Ir(III) Tetrazole Complexes within Ceramic Silica Nanophases for Photo-Induced Self-Marker and Antibacterial Application
Source: Nanomaterials (Basel). 2020 May 27;10(6):1020. doi: 10.3390/nano10061020 (PMC7352244; doi:10.3390/nano10061020)
Supplement: Supplementary file 1 [file nanomaterials-10-01020-s001.pdf]

nanomaterials

MDPI

Supplementary Materials

Use of cotton textiles coated by Ir(III) tetrazole complexes within ceramic silica nanophases for photo-induced self-marker and antibacterial application

Ilaria Zanoni[a,b], Magda Blosi[a*], Valentina Fiorini[c], Matteo Crosera[d], Simona Ortelli[a], Stefano Stagni[c], Alessandra Stefan[c], Sotiris Psilodimitrakoupoulos[f], Emmanuel Stratakis[f], Francesca Larese Filon[b], Anna Luisa Costa[a]

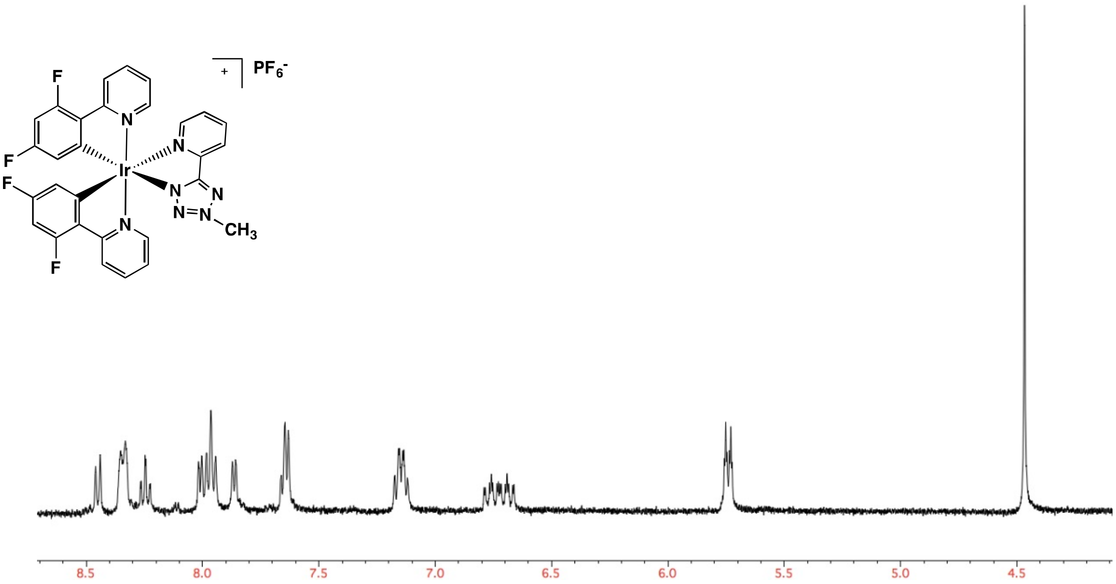

[F_2IrPTZ-Me]$^+$ ^{1}H-NMR (CD_3CN, 400 MHz) δ (ppm): 8.46-8.44 (d, 1H, J_{H-H} = 8.00 Hz); 8.36-8.33 (d, 2H, J_{H-H} = 8.80 Hz); 8.26-8.23 (m, 1H); 8.02-7.95 (m, 3H); 7.87-7.86 (d, 1H, J_{H-H} = 5.20 Hz); 7.66-7.63 (m, 2H); 7.17-7.12 (m, 2H); 6.79-6.69 (m, 2H); 5.75-5.73 (m, 2H); 4.47 (s, 3H).

Figure S1. ^{1}H NMR of **[F_2IrPTZ-Me]$^+$**, CD_3CN, 400 MHz.

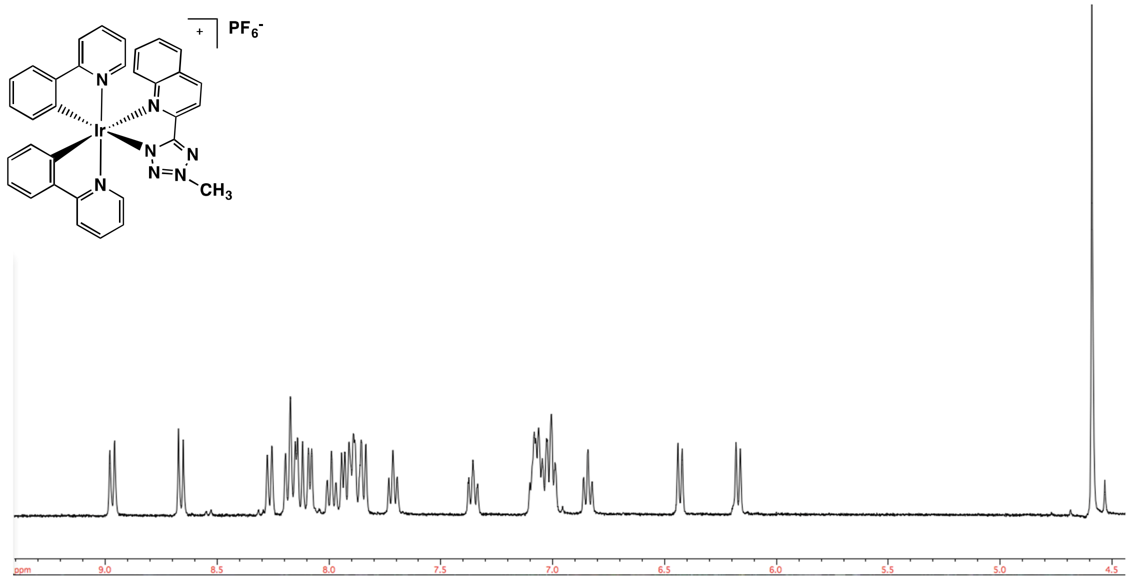

[IrQTZ-Me]+ ^{1}H-NMR (Acetone-d^6, 400 MHz) δ (ppm): 8.98 (d, 1H, J_{H-H} = 8.39 Hz), 8.67 (d, 1H, J_{H-H} = 8.39 Hz), 8.27 (d, 1H, J_{H-H} = 7.99 Hz), 8.19 – 8.07 (m, 4H), 8.01 – 7.83 (m, 5H), 7.73 (m, 1H), 7.37 (m, 1H), 7.10 – 6.98 (m, 5H), 6.86 (m, 1H), 6.44 (d, 1H, J_{H-H} = 7.59 Hz), 6.18 (d, 1H, J_{H-H} = 7.59 Hz), 4.59 (s, 3H).

Figure S2. ^{1}H NMR of **[IrQTZ-Me]+**, CD_3CN, 400 MHz.

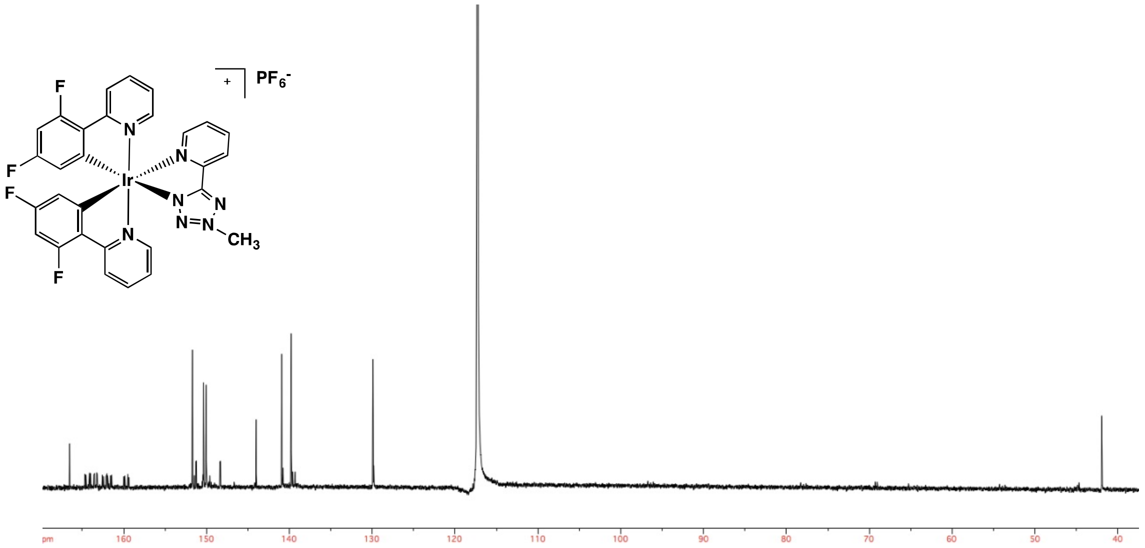

[F_2IrPTZ-Me]$^+$ ^{13}C-NMR (CD_3CN, 100 MHz) δ (ppm): 166.54, 164.69, 164.57, 164.13, 164.00, 163.60, 163.53, 163.27, 163.20, 162.59, 162.08, 162.02, 161.95, 161.47, 159.88, 159.50, 151.76, 151.71, 151.47, 151.30, 151.23, 150.43, 150.37, 150.05, 149.97, 149.61, 148.40, 148.32, 144.02, 140.94, 140.80, 140.77, 139.80, 139.62, 139.36, 139.32, 129.93, 129.83, 128.20, 124.84, 124.77, 124.74, 42.08.

Figure S3. ^{13}C NMR of **[F_2IrPTZ-Me]$^+$**, CD_3CN, 400 MHz.

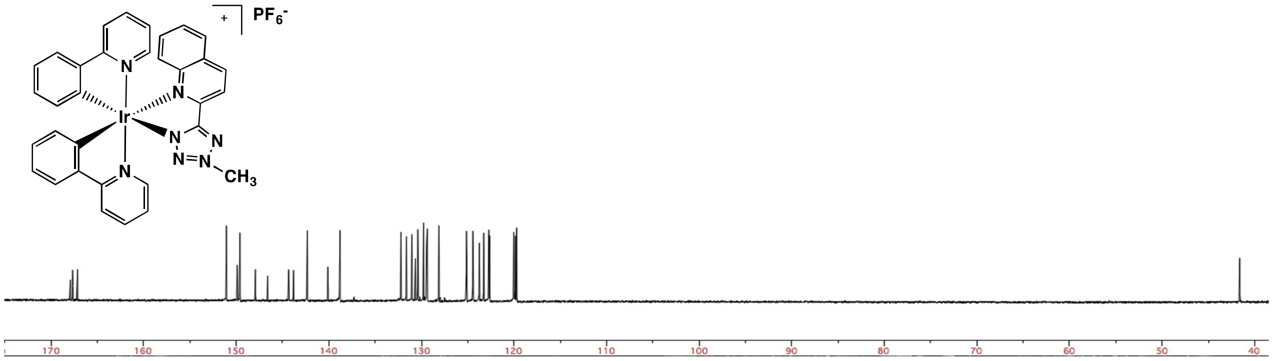

[IrQTZ-Me]$^+$ ^{13}C-NMR (Acetone-*d*^{6}, 100 MHz) δ (ppm) = 167.93 (Ct), 167.69, 167.15, 151.08, 149.91, 149.61, 147.95, 146.63, 144.35, 143.83, 142.34, 140.12, 138.84, 138.80, 132.22, 131.63, 131.04, 130.66, 130.40, 129.78, 129.46, 129.37, 128.12, 125.15, 124.46, 123.76, 123.28, 122.73, 122.63, 120.04, 119.87, 119.72, 41.62.

Figure S4. ^{13}C NMR of **[IrQTZ-Me]$^+$**, CD_3CN, 400 MHz.

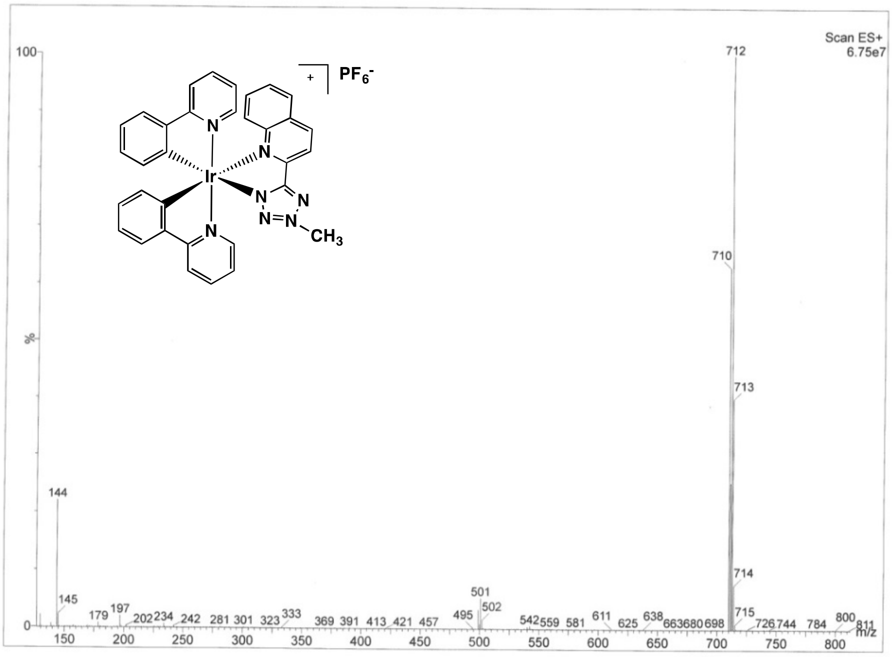

[IrQTZ-Me]$^+$ ESI-MS (*m*/*z*): [M$^+$] = 712 m/z [M$^-$] = 145 m/z (PF_6). Anal. Calcd. For $C_{33}H_{25}F_6IrN_7P$ (856.77) C 46.26, H 2.94, N 11.44. Found: C 40.95, H 2.85, N 9.55.

Figure S5. ESI-MS spectrum of **[IrQTZ-Me]$^+$** (positive ions region) [M]$^+$ = 712 *m*/*z*, CH_3CN.

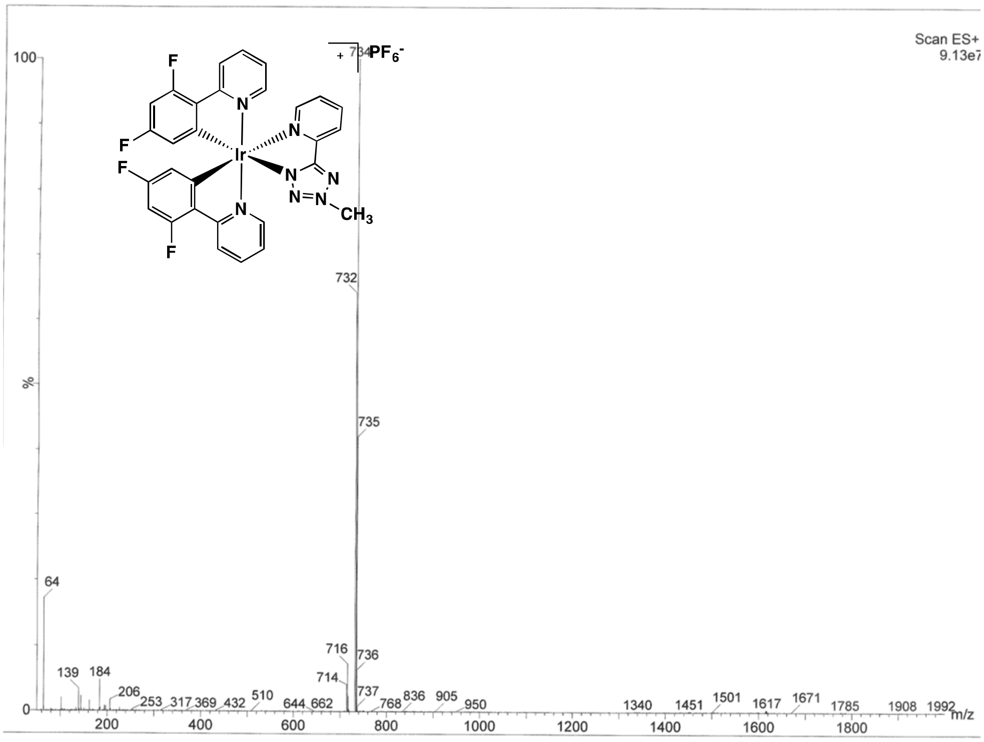

[F$_2$IrPTZ-Me]$^+$ ESI-MS (*m*/*z*): [M] $^+$ = 734 m/z [M] $^-$ = 145 m/z (PF_6). Anal. Calcd. For $C_{31}H_{23}Cl_4F_{10}IrN_7P$ (1048.53) C 35.51, H 2.21, N 9.35. Found: C 35.73, H 2.46, N 9.93.

Figure S6. ESI-MS spectrum of **[F$_2$IrPTZ-Me]$^+$** (positive ions region) [M]$^+$ = 734 *m*/*z*, CH_3CN.

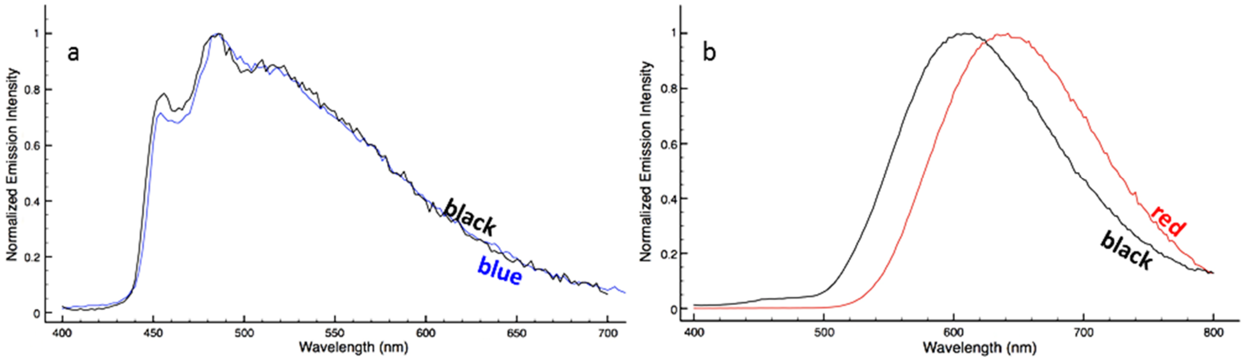

Figure S7. Comparison between normalised emission profiles **[F$_2$IrPTZ-Me]$^+$** (blue line) and **[IrQTZ-Me]$^+$** (red line) complexes and their **Ir@SiO$_2$ systems** (black lines).

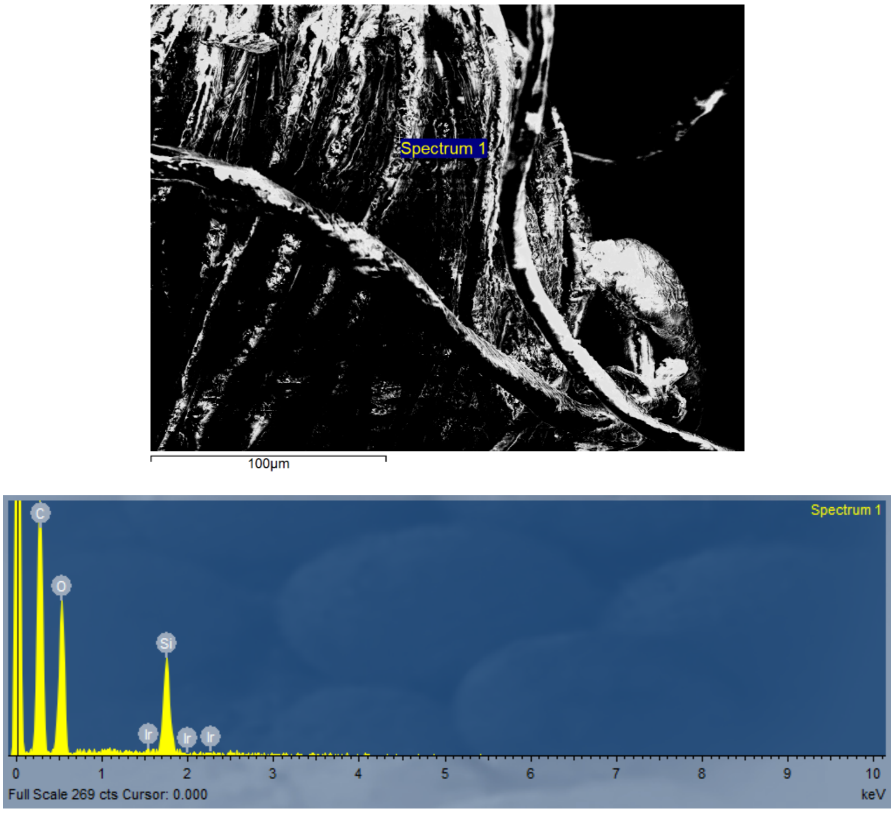

Figure S8. EDX spectrum of blue Ir@SiO_2 coated textile.

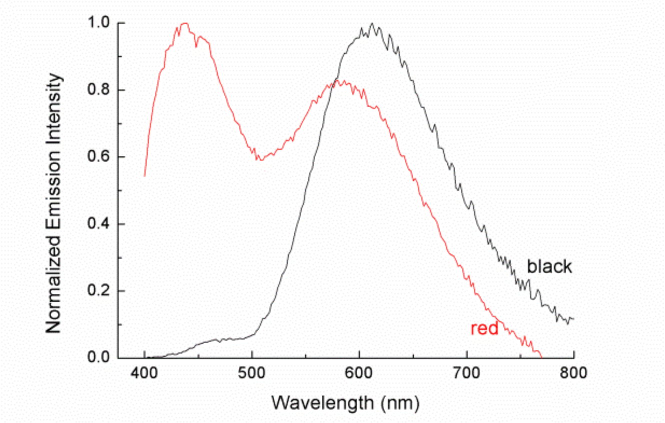

Figure S9. Comparison between normalised emission profile of **red Ir@SiO_2** nanosol system (black) and once used as coating on cotton textile (red).

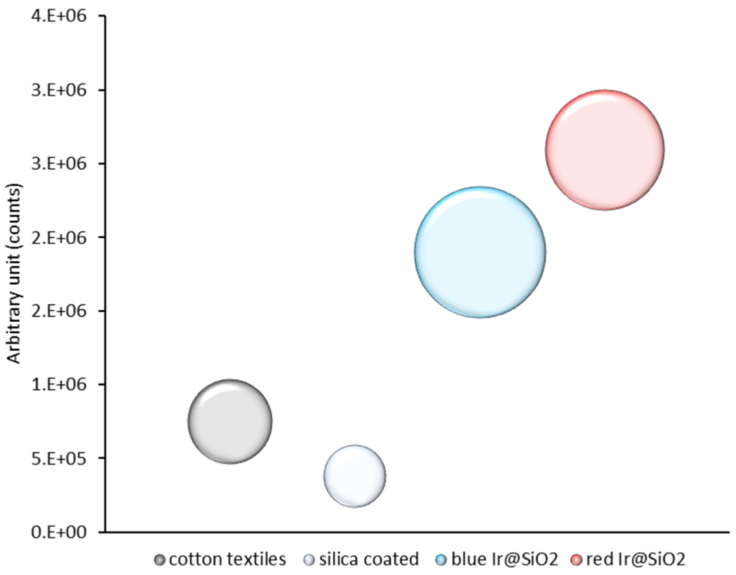

Figure S10. Qualitative evaluation of 2p-fluorescen images made by ImageJ on untreated cotton textiles and samples of cotton textile coated by silica, **blue Ir@SiO2 system** and **red Ir@SiO2 system**.

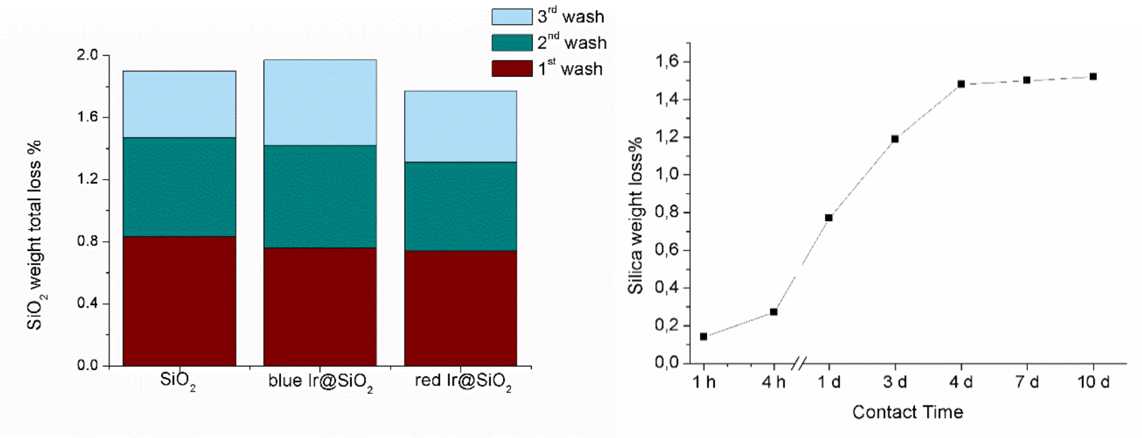

Figure S11. Release tests results collected both in dynamic (left) and static condition (right) for textiles coated by three-layer of **blue Ir@SiO2 system**.

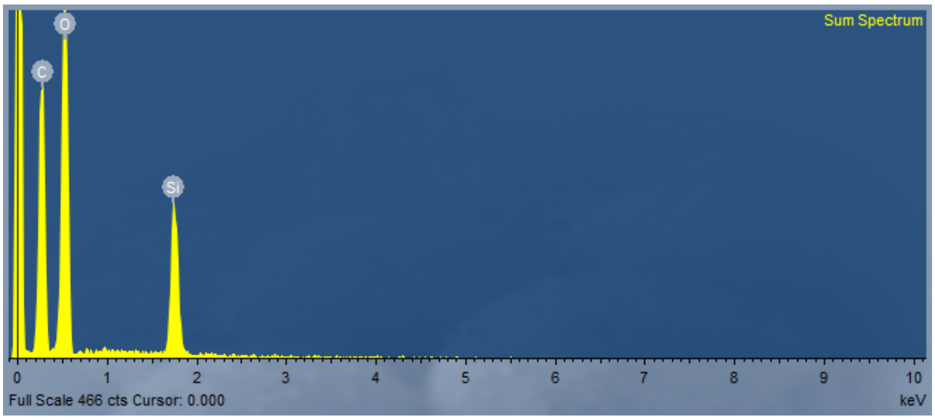

Figure S12. EDX spectrum of blue Ir@SiO_2 coated textile.

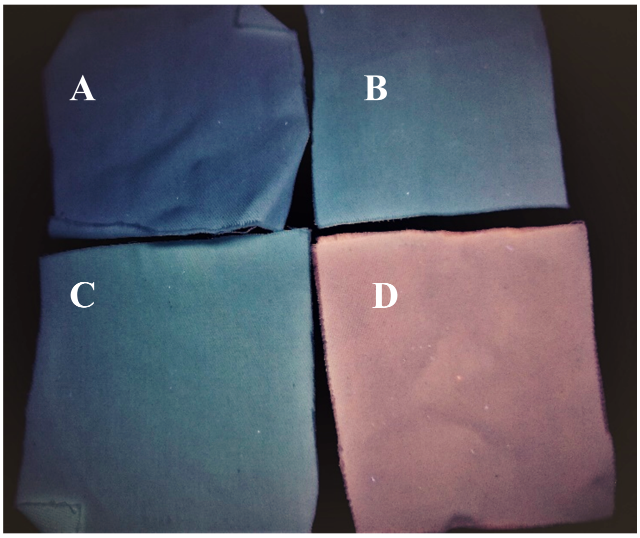

Figure S13. Real image of fabric samples under UV lamp (λ_{em}= 365 nm): silica coated (A), blue Ir@SiO_2 coated after 10 days of washing test in static condition (B), blue Ir@SiO_2 coated after 2 years (C) and red Ir@SiO_2 coated after 2 years (D) cotton textiles.

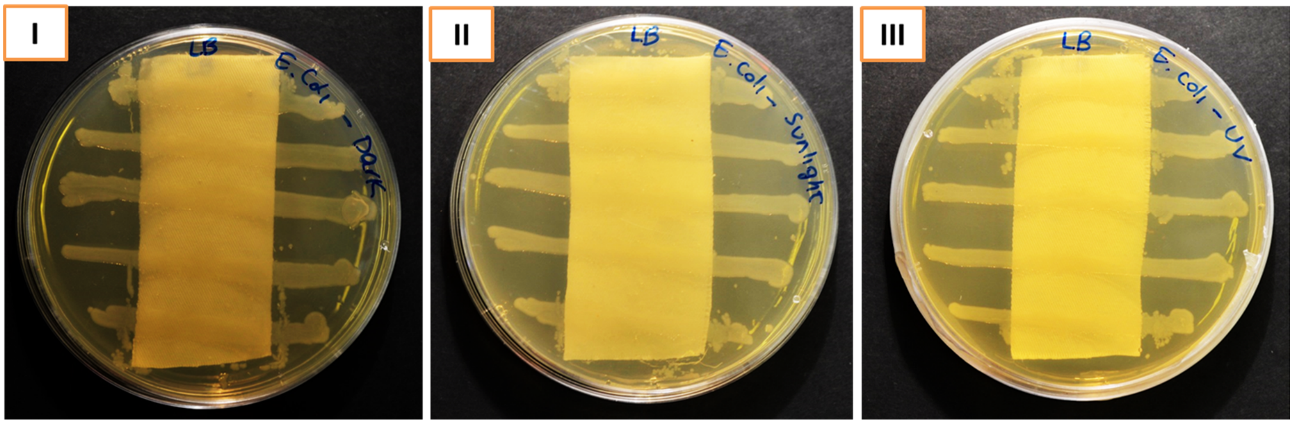

Figure S14. Image of AATCC 147 tests on red Ir@SiO_2 coated textiles once exposed in dark (I), natural light (II) and natural light + UV (III) conditions.

Table S1. Spectrometric relevant data, expressed as absorption and emission, of cationic Ir(III) complexes and Ir@SiO_2 nanosols systems.

CH_2Cl_2 as the solvent	**Absorption** λ_{abs}(nm);($10^{-4}\varepsilon$) ($M^{-1}cm^{-1}$)	**Emission 298 K**[a,b] λ_{em} (nm)	τ_{air} (µs)	τ_{Ar} (µs)	φ_{air} (%)	φ_{Ar} (%)	**Emission 77K**[c] λ_{em} (nm)	τ (µs)
**[IrQTZ-Me]$^+$**	253 (4.25), 310 (1.41), 374 (0.78)	638	0.220	0.550	2.8	4.5	568	1.56
**[F_2IrPTZ-Me]$^+$**	257 (6.24), 318 (2.70), 351 (1.20)	454, 486, 526	0.040	0.140	1.7	4.7	448, 480	6.62
H_2O as solvent	**Absorption** λ_{abs}(nm)	λ_{em} (nm)	τ_{air} (µs)	τ_{Ar} (µs)	φ_{air} (%)	$\Delta\lambda$ (nm)		
**Red Ir@SiO_2**	252, 312	610	0.100	n.d.*	n.d.*	28	n.d.*	n.d.*
**Blue Ir@SiO_2**	263, 286, 310	454, 484, 520	0.010	n.d.*	n.d.*	0	n.d.*	n.d.*

[a]:"Air" refers to air equilibrated solutions, "Ar" refers to deoxygenated solutions under argon atmosphere; [b]: $[Ru(bpy)_3]Cl_2/H_2O$ solution was used as reference standard for quantum yield determinations ($\Phi_r = 0.028$); [c]: in frozen CH_2Cl_2; *n.d. = not determined.

Table S2. SiO_2 weight loss% data relative to release test in dynamic condition (test for three layers coated textiles).

**Coating type (3 layers)**	**Dynamic release test** N° washing	**SiO_2 weight loss%**	**SiO_2 weight total loss%**	**Ir complex weight loss%**
**SiO_2**	1	0.83 ± 0.12	**1.90 ± 0.45**	/
	2	0.64 ± 0.08		/
	3	0.43 ± 0.03		/
**Blue Ir@SiO_2**	1	0.76 ± 0.09	**1.97 ± 0.52**	*
	2	0.66 ± 0.07		*
	3	0.55 ± 0.04		*
**Red Ir@SiO_2**	1	0.74 ± 0.14	**1.65 ± 0.66**	*
	2	0.57 ± 0.47		*
	3	0.46 ± 0.20		*

* values not detectable with ICP-OES analysis (< 0.01 ppm).

Table S3. Cumulative SiO_2 weight loss% data relative to release test in static condition for **blue Ir@SiO_2** coating (test for three layers coated textiles).

Contact Time	SiO_2 weight loss%	Ir weight loss%*
1 h	0.14 ± 0.03	-
4 h	0.27 ± 0.07	-
1 d	0.77 ± 0.06	-
3 d	1.19 ± 0.06	-
4 d	1.48 ± 0.02	-
7 d	1.50 ± 0.06	-
10 d	1.52 ± 0.07	-

* values not detectable with ICP-OES analysis (< 0.01 ppm).

Photophysical characterization. The wavelengths for the emission and excitation spectra were determined using the absorption maxima of the MLCT transition bands (emission spectra) and at the maxima of the emission bands (excitation spectra). Quantum yields (Φ) were determined using the optically dilute method by Crosby and Demas[1] at excitation wavelength obtained from absorption spectra on a wavelength scale [nm] and compared to the reference emitter by the following equation:

$$\phi_s = \phi_r \left[\frac{A_r(\lambda_r)}{A_s(\lambda_s)}\right] \left[\frac{I_r(\lambda_r)}{I_s(\lambda_s)}\right] \left[\frac{n_s^2}{n_r^2}\right] \left[\frac{D_s}{D_r}\right]$$

where A is the absorbance at the excitation wavelength (λ), I is the intensity of the excitation light at the excitation wavelength (λ), n is the refractive index of the solvent, D is the integrated intensity of the luminescence, and Φ is the quantum yield. The subscripts r and s refer to the reference and the sample, respectively. A stock solution with an absorbance > 0.1 was prepared, then two dilutions were obtained with dilution factors of 20 and 10, resulting in absorbance of about 0.02 and 0.08 respectively. The Lambert-Beer law was assumed to remain linear at the concentrations of the solutions. The degassed measurements were obtained after the solutions and bubbled for 10 minutes under Ar atmosphere, using a septa-sealed quartz cell. Air-equilibrated $[Ru(bpy)_3]Cl_2/H_2O$ solution (Φ = 0.028)[2] was used as reference. The quantum yield determinations were performed at identical excitation wavelengths for the sample and the reference, therefore deleting the I(λr)/I(λs) term in the equation. Emission lifetimes (τ) were identified by using the single photon counting technique (TCSPC) with the same Edinburgh FLSP920 spectrometer using pulsed picosecond LED (EPLED 360, FWHM < 800ps) as the excitation source, with repetition rates between 1 kHz and 1 MHz, and the above-mentioned R928P PMT as detector. The goodness of fit was assessed by minimizing the reduced $\chi 2$ function and by visual inspection of the weighted residuals. To record the 77 K luminescence spectra, the samples were put in quartz tubes (2 mm diameter) and inserted in a special quartz Dewar filled with liquid nitrogen. The solvent used in the preparation of the solutions for the photophysical investigations was of spectrometric grade.

References

1. Crosby, G.A.; Demas, J.N. Measurement of photoluminescence quantum yields. Review. *J. Phys. Chem.* **1971**, *75*, 991–1024, doi:10.1021/j100678a001.
2. Nakamaru, K. Synthesis, Luminescence Quantum Yields, and Lifetimes of Trischelated Ruthenium(II) Mixed-ligand Complexes Including 3,3′-Dimethyl-2,2′-bipyridyl. *Bull. Chem. Soc. Jpn.* **1982**, *55*, 2697–2705, doi:10.1246/bcsj.55.2697.
